# Concurrent intracranial tuberculomas and spinal tuberculosis with multifocal abscesses: a rare case report

**DOI:** 10.1097/MS9.0000000000003024

**Published:** 2025-02-07

**Authors:** Alok Chandra Thakur, Gopal Sedain, Sangit Chhantyal, Amit Kumar Mishra, Bikram Prasad Gajurel

**Affiliations:** a Department of Neurosurgery, Tribhuvan University Teaching Hospital, Kathmandu, Nepal; b Maharajgunj Medical Campus, Tribhuvan University, Institute of Medicine, Kathmandu, Nepal; c Department of Neurology, Tribhuvan University Teaching Hospital, Kathmandu, Nepal

**Keywords:** abscesses, extrapulmonary, neurotuberculosis, seizures, tuberculomas

## Abstract

**Introduction:**

Neurotuberculosis is a severe form of tuberculosis affecting the central nervous system, manifesting as tuberculomas or abscesses. Simultaneous intracranial and spinal tuberculomas with abscesses are exceptionally rare, presenting unique diagnostic and therapeutic challenges.

**Case presentation:**

A 40-year-old female presented with headaches, seizures, and lower limb weakness. The magnetic resonance imaging (MRI) revealed multiple intracranial and spinal tuberculomas with abscesses. She was treated with antitubercular therapy and corticosteroids, along with surgical drainage, leading to significant improvement.

**Discussion:**

Neurotuberculosis can present without evident pulmonary involvement, making early diagnosis crucial. MRI plays a key role in identifying tuberculomas, which must be differentiated from other lesions such as neurocysticercosis or gliomas. Conservative management with ATT and steroids is often effective, particularly in resource-limited settings. Surgical intervention is reserved for cases with severe neurological deficits or diagnostic uncertainty.

**Conclusion:**

This case highlights the need to consider neurotuberculosis in patients with neurological symptoms, especially in endemic areas. The rarity of concurrent intracranial and spinal tuberculomas with abscesses requires a multidisciplinary approach for early diagnosis and effective management.

**HIGHLIGHTS**

- Up to one-third of neurotuberculosis cases lack extra-neural signs, making it essential to consider TB even without such evidence.
- MRI is a key non-invasive tool for diagnosing tuberculomas and distinguishing them from similar conditions like neurocysticercosis or glioma.
- Treatment primarily involves ATT and steroids, with surgery reserved for severe cases, emphasizing the need for timely intervention to prevent lasting neurological damage.

## Introduction

According to the Global tuberculosis report 2022, an estimated 10.6 million people fell ill with tuberculosis (TB) worldwide, with a total of 1.3 million deaths ^[1]^. TB is the second leading cause of infectious disease related mortality ^[1]^. It primarily infects lungs (Pulmonary TB), however, may involve other organs causing extrapulmonary TB^[2]^. Neurological involvement is the most serious and deadly manifestation of extrapulmonary TB^[3]^.

Central nervous system (CNS) TB comprises about 1–5% of total TB cases and 10% of AIDS-related TB cases^[4]^, typically diagnosed in the third to fifth decades of life^[5]^. It can be intracranial or spinal TB^[6]^. Intracranial TB may manifest as tuberculous meningitis, tuberculous encephalopathy, tuberculoma and tuberculous abscess^[7]^. Spinal TB can be extradurally manifested as tuberculous spondylitis, paraspinal and epidural abscess, and intradural encompassing spinal arachnoiditis, spinal tuberculoma, and tuberculous myelitis^[4]^.

Among CNS TB, intracranial type is far more common than spinal with a ratio of about 30–40:1^[5]^. The cases with concomitant intracranial and spinal involvement is extremely rare^[8]^. In a study based in India, 6.15 % of 306 CNS TB cases had concomitant pathology^[7]^. Here, we report a case of concurrent intracranial tuberculomas in cerebrum and cerebellum, and epidural, prevertebral, paravertebral and bilateral psoas abscesses. This, to the best of our knowledge, has not been reported so far. This case has been reported in line with the SCARE 2023 criteria^[9]^.

## Case presentation

A 43-year-old male, a bus driver by occupation, presented with a 6-month history of abnormal body movements. His first episode involved twisting, mouth frothing, teeth clenching, eye rolling, and unconsciousness, followed by postictal confusion. A similar episode occurred shortly after, leading to his admission and initiation of antiepileptic medication. After 2 months without symptoms, he experienced jerky movements starting from the left toe and spreading to the shoulder, with mouth deviation, though his consciousness remained intact.These episodes recurred weekly. He also reported low back pain, progressive weakness in his lower limbs, difficulty standing and walking, fever, anorexia, and weight loss. His brother has a history of seizure disorder. The patient has no other comorbid conditions and is an occasional smoker.

Examination revealed intact higher mental function, cranial nerves, cerebellum and no signs of meningeal irritation. Strength was 4⁄5 on the left upper and left lower limb muscles. All deep tendon reflexes and plantar reflexes were normal bilaterally. All sensations were intact.

**Figure 1.**
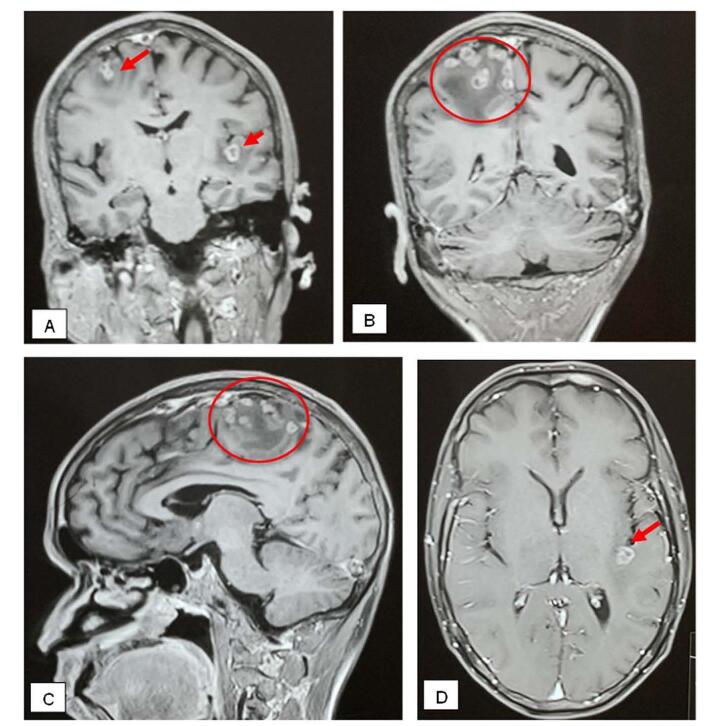
T1 weighted MRI of brain: (A & B) oblique coronal cut, (C) sagittal cut, and (D) axial cut showing features of multiple tuberculomas with associated edema in cerebrum (arrows and rings).

**Figure 2.**
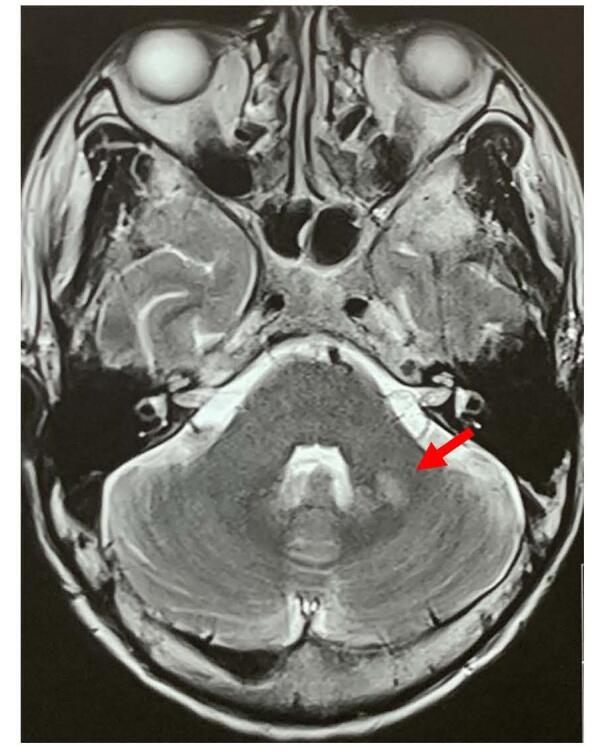
T1 weighted MRI of brain axial cut showing features of multiple tuberculomas in cerebellum (arrow).

**Figure 3.**
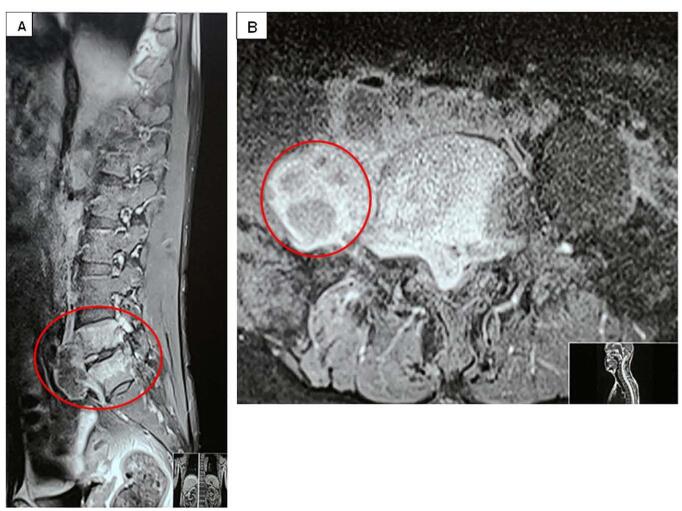
T2 weighted MRI of spine: (A) sagittal cut and (B) axial cut showing signal changes in the L5 vertebral body and multifocal abscesses (rings).

Ultrasonography of the neck showed multiple conglomerated lymph nodes, few round, few oval in left level IV, few with loss of fatty hilum, features likely of cervical lymphadenopathy, probably tubercular. The magnetic resonance imaging (MRI) of brain showed multiple small ring enhancing discrete and conglomerated lesions in bilateral cerebral hemisphere and cerebellum with significant edema-features likely of Tuberculomas (Figs. 1 and 2). The MRI of spine showed T2 high signal intensity in marrow, signal changes in the L5 vertebral body and multiple pockets of peripheral enhancing T2 high signal intensity collection in epidural, prevertebral, paravertebral and bilateral psoas muscle lesion in L5 and S1 level causing severe ventral spinal canal stenosis with compression of cauda equina-features suggestive of infective pathology, likely tubercular (Fig. 3). He was negative for HIV and syphilis. AFB was not detected in sputum and his chest x-ray was normal. Ultrasound-guided aspiration and drainage of the psoas abscess were performed, and the pus obtained was sent for investigation. The findings revealed acid-fast bacilli on Ziehl-Neelsen staining, and *Mycobacterium tuberculosis* (MTB) was detected using the GeneXpert assay, confirming the diagnosis of tuberculosis. A final diagnosis of focal onset motor seizure with preserved awareness, secondary to tuberculoma, with tubercular abscesses in the epidural, prevertebral, paravertebral, and bilateral psoas muscles at the L5 and S1 levels, associated with L5-S1 spondylodiscitis and cauda equina syndrome, was made.

He was started on four anti-tubercular drugs (isoniazid, rifampicin, pyrazinamide, ethambutol) along with pyridoxine and two anti-epileptic drugs (levetiracetam, clobazam). He was also treated with intravenous dexamethasone 6 mg thrice daily for 13 days, followed by oral dexamethasone in a tapering regimen, starting at 6 mg thrice daily and gradually reduced to 0.5 mg per day over 6 months. The treatment was well-tolerated, with no significant adverse reactions.

Three months after the start of treatment, the patient underwent surgical decompression and posterolateral fusion for L5-S1 spondylodiscitis. The excised biopsy sample revealed a necrotizing granulomatous lesion. The four-drug therapy was given for two months followed by three drugs (isoniazid, rifampicin, ethambutol), which was planned for the next 16 months.

**Figure 4.**
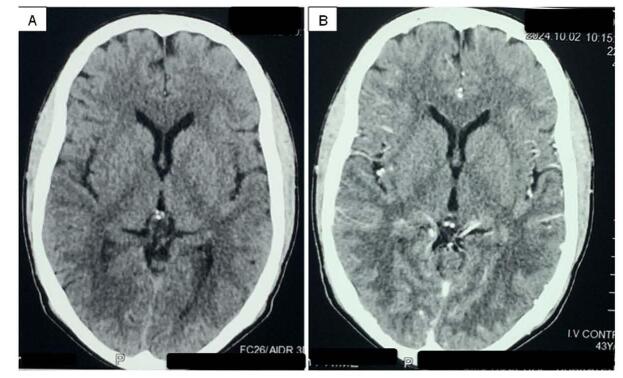
(A) Non-contrast CT and (B) contrast enhanced CT brain at six months follow up showing no tuberculoma.

**Figure 5.**
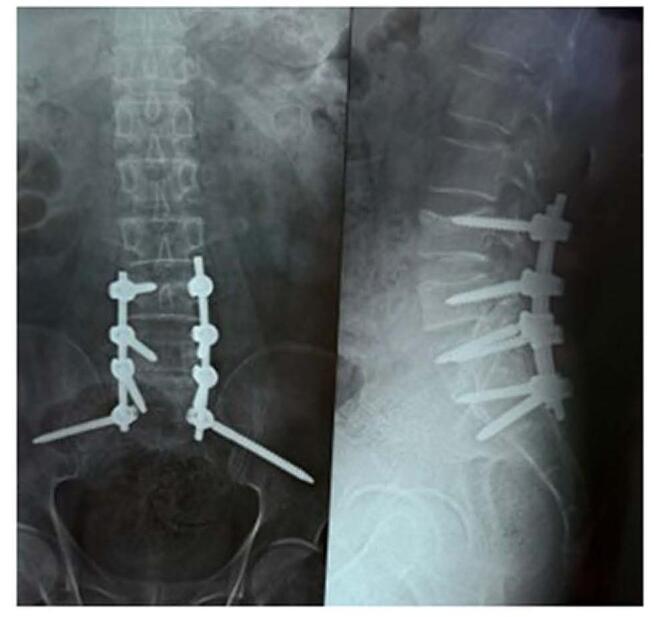
X-ray spine antero-posterior and lateral views at follow up showing implants in situ.

At the 6-month follow-up, the patient showed consistent improvement in power. He was advised Computed Tomography scan of brain showing no tuberculoma (Fig. 4) and plain X-ray of spine showing implants in-situ (Fig. 5).

## Discussion

Even in TB-endemic areas, CNS TB is an uncommon occurrence^[10]^. 10% of patients with systemic TB experience CNS involvement as a result of hematogenous dissemination from the initial focus, which is typically the lungs^[11]^. Young people in impoverished nations are primarily affected by primary neurotuberculosis. According to the literature, there is a minor male predilection for intramedullary TB (M:F = 1.5:1) and 50% of cases with CNS involvement appear to be under 10 years old^[10,12]^.

Similar to the case described by Thacker and Puri, our patient had primary tuberculosis of the central nervous system, affecting the cerebellum, extradural spine, and cerebrum^[11]^.Cases by Kulkarni *et al*^[13]^ Krishnan *et al*^[14]^, and Chitre *et al*^[15]^, all had extra-neural TB symptoms or were undergoing treatment for tuberculous meningitis.

On the other hand, extra-neural tuberculosis evidence may be absent in as many as one-third of neurotuberculosis cases. Thus, the likelihood of tuberculous etiology should not be ruled out in the absence of an extra-neural cause, as in our instance. Our patient, a 43-year-old man from an area where tuberculosis is widespread, initially displayed symptoms of compressive myelopathy and convulsions. Using MRI, a conclusive diagnosis of concurrent intracranial and spinal tuberculoma was made. With ATT and steroids, his neurological recovery was complete. It was determined to be a primary case of neurotuberculosis in an immunocompetent patient because there was no evidence of extra-neural TB or immunosuppression.Eleven adult and five juvenile instances (≤12 years) with coexisting intracranial and intraspinal tuberculoma were documented in a literature study conducted by Ghane *et al*^[16]^. Our patient’s age and the clinical manifestation of compressive myelopathy alone were comparable to those of instances reported by Bansal *et al*^[17]^ and Thacker and Puri^[11]^. Our case was quite similar to the instances reported by Krishnan *et al*^[18]^ and Chitre *et al*^[15]^, who both exhibited compressive myelopathy characteristics with intracranial symptoms.An intrusive process like a biopsy or open surgery is not necessary when using the sensitive, noninvasive MRI for tuberculoma localization and diagnosis^[19,20]^. However, our patient needed a tissue diagnosis to guide anti-microbial therapy.

In radiological terms, neurocysticercosis, glioma, ependymoma, fungal infections, toxoplasmosis, and lymphoma would be the differential diagnosis for intramedullary spinal tuberculomas or lesion other than tuberculomas. Nonetheless, adults frequently have these lesions^[21]^ The cornerstone of treatment is conservative control with ATT and steroids as anti-edema medicines^[17]^. Conservative medical therapy is a viable, affordable, safe, and effective choice, particularly in resource-poor and developing nations^[22]^. WHO and national tuberculosis treatment guideline of Nepal recommend 9–12 months of treatment for TB meningitis (2HRZE/7-10HRE) along with steroids and antiepileptics^[23,24]^. However our patient was planned for an 18-month treatment course due to the severity of the disease involvement. Although their effectiveness in treating patients with perilesional edema is mainly unknown, steroids nonetheless have a role. Patients who have huge lesions that significantly compress their body, who do not respond clinically or radiologically, who worsen under conservative treatment, or who have an unclear diagnosis may consider surgery. Early surgical decompression is necessary for patients with severe neurological abnormalities since postponement can damage the spinal cord irreversibly and result in lifelong neurological consequences^[21]^.

## Conclusion

This case underscores the rarity of concurrent intracranial and spinal tuberculosis in immunocompetent patients without extra-neural involvement, highlighting the need to consider neurotuberculosis even without pulmonary signs. The successful outcome with anti-tubercular therapy and steroids emphasizes the importance of early diagnosis and conservative management in preventing severe neurological damage.

## Data Availability

Datasets generated during and/or analyzed during the current study are publicly available.
